# Evaluation of Problematic Media Use in Children with Cerebral Palsy Aged 4–11 Years: A Case–Control Study

**DOI:** 10.3390/children12060675

**Published:** 2025-05-24

**Authors:** Orhan Coşkun, Abdurrahman Zarif Guney, Uğur Topçu, Mustafa Karaduman

**Affiliations:** 1 Department of Pediatric Neurology, Gaziosmanpasa Training and Research Hospital, University of Health Sciences, Istanbul 34090, Turkey; dr.orhancoskun@hotmail.com; 2 Cerrahpaşa Faculty of Medicine, Istanbul University-Cerrahpasa, Istanbul 34090, Turkey; 3 Department of Pediatrics, Gaziosmanpasa Training and Research Hospital, University of Health Sciences, Istanbul 34090, Turkey; drugurtopcu@gmail.com (U.T.); mustafa_karadumann@outlook.com (M.K.)

**Keywords:** problematic media use, cerebral palsy, screen exposure

## Abstract

**Background/Objectives**: Excessive screen exposure that negatively impacts a person’s life is referred to as problematic media use (PMU). In healthy children, PMU is associated with a sedentary lifestyle and negative health outcomes. However, this issue is not well understood in children with cerebral palsy (CP). The aim of our study was to evaluate PMU and screen exposure times in children with CP. **Methods**: All CP patients with variable etiology who were followed up at the child neurology clinic and met the inclusion criteria were included in our study. A total of 91 patients diagnosed with CP and 92 control children, aged 4–11, participated in the study. Daily screen exposure times, sleep durations, and body mass index (BMI) were evaluated. In children with CP, additional data such as the need for special education, CP subtype, epilepsy status, and mobility status were recorded. All participants were administered the long form of the Problematic Media Use Scale, which is valid for children aged 4–11. **Results**: When comparing screen exposure between the patient group and the control group, the patient group had significantly higher screen exposure (*p* < 0.001). Spearman’s rho correlation coefficients, calculated to evaluate the relationship between age, sleep duration, screen exposure time and screen addiction questionnaire scores of the children in the patient and control groups, were statistically significant. For the patient group specifically, there was a significant positive relationship between screen exposure duration and screen addiction questionnaire scores (r = 0.380, *p* < 0.001). **Conclusions**: In our study, it was seen that problematic media use was no different in CP patients within the same age group. However, screen exposure durations were found to be higher than in the normal population.

## 1. Introduction

CP is a group of permanent movement and posture disorders caused by non-progressive disturbances in the developing fetal or infant brain. It affects muscle tone, coordination, balance, and motor skills, and often leads to limitations in functional abilities. While the brain injury that causes CP does not worsen over time, the symptoms may change as the child grows. In addition to motor impairments, individuals with CP may also experience associated conditions such as intellectual disabilities, epilepsy, sensory impairments, communication difficulties, and behavioral challenges. The severity and combination of symptoms vary widely among individuals, making cerebral palsy a highly heterogeneous condition [1].

Many factors such as prematurity, congenital anomalies, hypoxic ischemic encephalopathy, genetic factors, and congenital infections are blamed for the etiology of cerebral palsy, but it often occurs due to multifactorial causes [2]. CP is accompanied by cerebral dysfunctions such as sensory or perceptual changes, intellectual disability, communication and behavioral disorders, and motor movement disorders. Chronic pain occurs in 50% to 75% of individuals, intellectual disability in 50%, speech–language disorders in 40% to 60%, visual impairment in 30% to 50%, behavioral disorders in 25% to 40%, sleep disorders in 20%, and hearing impairment in 10% to 20% [3,4].

Excessive screen exposure that negatively affects a person’s life is called PMU [5]. This type of use is quite common, especially in children and adolescents. Examples of PMU include television addiction, smartphone addiction, computer addiction, and digital game addiction [6]. Increased screen exposure in children is associated with a sedentary lifestyle and negative health outcomes. The American Academy of Pediatrics (AAP) recommends that children under the age of 2 should not be exposed to screens, but this is not very possible today. The American Academy of Pediatrics has also stepped back from its screen time guidelines for this reason. The new approach aims to replace screen time with social activities [7]. Additionally, research indicates that boys tend to spend more time in front of screens than girls, and adolescents are exposed to screens more than younger children. In the study, children from primary to high school levels were classified. It was found that the total time spent on digital gaming, social media, browsing websites, and watching TV or movies increased with age. High school students had the highest usage times. [8]. The issue of PMU has not been extensively studied in children with CP. The aim of our study was to evaluate media use and screen time in these children. Given that cerebral palsy can have negative effects on social development, we also aimed to explore the differences between children with CP and their typically developing peers aged between 4 and 11.

## 2. Materials and Methods

### 2.1. Design

Our study was designed as a single-center prospective case–control study. It included all children with cerebral palsy of various etiologies who were being followed at the Department of Pediatric Neurology at Gaziosmanpaşa Training and Research Hospital in Turkey. Demographic data were collected from both the cerebral palsy and control groups, as well as from their families. Additionally, daily screen time and sleep duration were evaluated. Body weight was measured using a digital physician’s scale, and height was measured with a stadiometer. To ensure consistency, the same equipment was used for all participants. Body mass index (BMI) was calculated from each child’s height and weight, and BMI standard deviation scores (SDS) were determined using reference data for Turkish children provided by Olcay Neyzi et al. [9].

For children with cerebral palsy, further information was recorded, including the need for special education, CP subtype, epilepsy status, and mobility level. The long form of the Problematic Media Use Scale, which is validated for children aged 4–11, was administered to all participants.

### 2.2. Participants

The study included patients followed up at the pediatric neurology clinic of Gaziosmanpaşa Training and Research Hospital, located in Istanbul, Turkey, while the control group was selected from the hospital’s pediatric clinic. The number of patients who were regularly followed up in our clinic was 167. Out of 167 regularly followed children with cerebral palsy, 128 were reached and agreed to participate in the study. Of these, 37 were excluded due to severe cognitive and motor impairments that prevented them from using visual media tools such as televisions or tablets. As a result, 91 children with cerebral palsy and 92 control children aged 4 to 11 were included in the study. The control group consisted of children aged 4 to 11 who presented to the general pediatric clinic in August 2024. Our study consisted of participant groups of Middle Eastern origin. The gender ratios of the control and patient groups were matched as closely as possible. The control group children had no chronic organic or neuropsychiatric disorders and were not on any long-term medication. All consenting controls were included in the study, regardless of their other characteristics. Enrollment of the control group was discontinued once the target number of patients was reached. In the patient group, children with chronic neuropsychiatric disorders or other chronic illnesses, except for cerebral palsy, were excluded from the study.

### 2.3. Long-Form Problematic Media Use Scale 

The scale, developed by Domoff et al. in 2017 to assess problematic media use (PMU) in children aged 4–11, includes a 27-item long form and a 9-item short form [5]. The Turkish validity and reliability study for both forms was conducted by Furuncu and Öztürk in 2019. The scale has a single-factor structure and uses a 5-point Likert scale for scoring (1 = never; 5 = always). The total PMU score is calculated by averaging the scores from all items. Higher scores on the scale indicate greater problematic media use [6].

### 2.4. Statistical Analysis

For evaluating the findings obtained in the study, IBM Scientific Package for Social Science (SPSS) package program (Version 23, Chicago, IL, USA, 2015) was used for statistical analysis. For evaluating the study data, in addition to descriptive statistical methods (mean, standard deviation, frequency), the independent samples *t*-test and one-way ANOVA test were used in comparisons of parameters showing a normal distribution, the Mann–Whitney U-test was used in comparisons of parameters not showing a normal distribution, and Spearman’s rank correlation test was used to measure the relationship between two parameters. For the comparison of categorical variables, the Chi-square test was used. Significance was evaluated at a *p* < 0.05 level.

## 3. Results

The Cronbach’s alpha reliability coefficient, commonly used to assess the reliability of scales and ensure consistent results across repeated measurements, was calculated for the screen addiction questionnaire. The scale demonstrated high reliability, with a coefficient of 0.85 (0.81 < α < 1.00). Demographic findings are shown in Table 1.

When comparing the patient group and the control group by gender, no statistically significant difference was found (*p* > 0.05). Similarly, there was no statistically significant difference between the two groups in terms of age and sleep duration (*p* > 0.05) (Table 2). However, comparing screen exposure time between the patient and control groups, the results showed statistically significant higher screen exposure in the patient group (*p* < 0.001) (Table 2).

The mean BMI SDS was found to be 0.12 in children with cerebral palsy and 0.13 in the healthy control group, with no statistically significant difference between the groups (*p* = 0.78). (Table 2).

The Spearman’s rho correlation coefficient was used to assess the relationship between age, sleep duration, screen exposure, and the scores from the problematic media use questionnaire among children in the patient and control groups [5]. The analysis found no statistically significant relationship between either sleep duration or screen exposure and the questionnaire scores (*p* > 0.05). However, a positive correlation was observed between age and problematic media use scores (r = 0.185, *p* = 0.012).

For the patient group, which had significantly higher screen exposure than the control group, a statistically significant positive correlation between screen exposure and questionnaire scores was identified (r = 0.380, *p* < 0.001).

A comparison of the total questionnaire scores between the patient and control groups revealed no significant difference (*p* > 0.05) (Table 3). Additionally, no statistically significant difference was found in the total scores between children with and without special educational needs, according to their perception status (*p* > 0.05) (Table 3). The same result was observed when comparing scores between children with and without epilepsy, as well as among children with different types of involvement (tetraparesis, diparesis, hemiparesis) (*p* > 0.05) (Table 3). Lastly, no significant difference was noted in total scores based on the mobility status of the patients (mobile, assisted mobile, immobile) (*p* > 0.05) (Table 3).

## 4. Discussion

Increased screen time in children can lead to problems like a sedentary lifestyle, weakened social relationships, and social difficulties.

A study conducted in 2022 reviewed 68 reports and various articles, revealing that digital media use is linked to a sedentary lifestyle, excessive junk food consumption, malnutrition, and obesity. Increased screen exposure also raises the risk of eye problems such as myopia, blurred vision, eye fatigue, ocular redness, dry eye disease, and headaches. Additionally, tooth decay related to high junk food intake has been observed [10]. It has also been reported that cardiovascular risk increases in individuals addicted to digital media. Studies have shown that excessive digital media use is associated with high blood pressure, low HDL cholesterol, increased sympathetic arousal, cortisol imbalance, and insulin resistance [11]. A review of the literature revealed that there are no existing studies on screen exposure in children with CP.

When the data from our study are examined, it is notable that there is no gender difference between the control group and the patient group. This is important, because screen exposure is generally higher in boys. It is also known that screen exposure tends to increase with age [8]. In our study, there was no statistically significant gender difference between the patient and control groups. Additionally, when both groups were evaluated, it was found that screen exposure duration increased with age, which is consistent with findings in the existing literature (*p* < 0.05).

Screen exposure is known to cause sleep problems. According to a report by Chassiakos et al., increased exposure to digital media and the use of televisions, computers, and mobile devices in the bedroom among young children were associated with reduced sleep duration [12]. In our study, no difference was observed in the average daily sleep duration between the patient and control groups. It was concluded that the sleep duration of our patients with cerebral palsy was not shorter than those of children in the same age group. As a result of the study, screen exposure duration in children with cerebral palsy was found to be statistically significantly higher compared with the control group (*p* < 0.05). However, this increased screen exposure did not lead to any reduction in sleep duration.

Since our patients had motor or cognitive difficulties, it was thought that they spent more time at home compared with other children. Therefore, children with cerebral palsy may be at a greater risk of problematic media use than their healthy peers. However, when assessed using the problematic media use questionnaire, no significant difference was found between the patient and control groups. The questionnaire includes items related to hiding or lying about media use. Because it contains questions about behaviors like deceiving family members or manipulating media usage, children with cerebral palsy—who may have limited ability in these areas—might not respond in the same way. This may explain the lack of difference between the groups. Nevertheless, the average daily screen exposure time was found to be higher in children with cerebral palsy.

We examined the patients by subgroups, as problematic media use might be higher in children who require special education due to impulsivity. All patients in the study were attending nursery, kindergarten, or primary school. In total, 60 of them were evaluated by psychiatrists and teachers as not needing additional learning support, while 31 were receiving special education. When these two groups were compared, no significant difference was found in terms of problematic media use. This suggests that perceptual delay in children with cerebral palsy does not pose an additional risk factor for problematic media use.

Epilepsy is one of the common problems in cerebral palsy [13]. In our study, patients with epilepsy were using one or two antiepileptic drugs, and their seizures were under control. Patients with drug-resistant epilepsy were excluded from the study, as they were not suitable for the problematic media use questionnaire and often had limited screen access by choice. No significant difference in problematic media use was found between the patients with epilepsy and those with cerebral palsy without epilepsy.

CP is a condition that affects mobility, and the main goal of treatment is to reduce the child’s functional disability. Various multidisciplinary approaches are used to address motor impairments, including spasticity treatments, orthotics, physical therapy, and orthopedic surgery [14]. Regardless of the method used, physical inactivity in the child reduces the likelihood of treatment success.

Our study, when screen exposure was evaluated according to the type of cerebral palsy and the child’s mobility status, no statistically significant difference was found. Initially, we expected problematic media use to be higher in non-mobile patients. However, the lack of difference suggests that these children are also socially integrated, which is a positive outcome. This helps minimize the negative effects of movement disorders, promotes socialization, and supports the development of self-esteem in children with CP [15].

In this study, the sample primarily included children with mild forms of cerebral palsy, as those with severe cognitive and motor impairments—who were unable to use visual media tools—were excluded. As a result, the study population consisted of individuals who were ambulatory and had preserved feeding abilities. Within this selected group, BMI SDS values were found to be comparable with those of healthy peers, with no significant differences observed between the groups. These findings suggest that children with milder forms of CP may maintain a nutritional status similar to that of typically developing children [16].

Our study is the first to evaluate problematic media use in children with cerebral palsy. Some patients were excluded because they were not suitable for assessment. Overall, the risk of problematic media use appears to be similar to that of the healthy population. However, screen exposure time was found to be longer in children with cerebral palsy.

Despite the increased screen time, the results of the problematic media use questionnaire, which was completed by the families, did not show significantly higher scores. This may be due to families normalizing excessive screen time or due to the children’s difficulties in expressing themselves, which could have influenced the responses.

Raising awareness about this issue is crucial. Children with cerebral palsy already face challenges in participating in social life, and problematic media use may further increase this disadvantage. It could also negatively impact their mobility and limit opportunities for physical activity and exercise.

Our study was designed as a case–control study. Future research could explore the effects of reducing screen exposure and screen addiction on mobility and cognitive functions. The long-term impact of these factors can also be monitored in follow-up studies.

## 5. Conclusions

It was seen that problematic media use was no different in cerebral palsy patients within the same age group. However, screen exposure times were found to be higher than in the normal population. It has also been observed that the need for special education, having epilepsy, mobility status, and the type of involvement do not create differences in problematic media use. Follow-up studies can be designed with larger patient groups. In these studies, the development of patients’ motor and cognitive skills over time can be examined by limiting problematic media use. Additionally, in an environment where screen use is so prevalent, there is clear potential to integrate it into patients’ treatments. More research is needed on this issue [17].

## Figures and Tables

**Table 1:** Demographic characteristics of the patient and control groups.

Total Number (N, %)	183 (100%)
**Groups (n, %)**	**183 (100%)**
Control	92 (50.27%)
Patient	91 (49.73%)
**Age (years)(mean ± SD)**	**8.12 ± 2.74**
Control	8.18 ± 2.29
Patient	8.65 ± 3.51
**Total gender (n, %)**	**183 (100%)**
Female	57 (31.15%)
Male	126 (68.85%)
**Control group gender (n, %)**	**92 (100%)**
Female	28 (30.43%)
Male	64 (69.57%)
**Patient group gender (n. %)**	**91 (100%)**
Female	29 (31.87%)
Male	62 (68.13%)
**Sleep duration (h) (mean ± SD)**	**9.53 ± 1.01**
Control	9.4 ± 1.09
Patient	9.66 ± 0.91
**Screen exposure (h) (mean ± SD)**	**2.97 ± 0.88**
Control	2.71 ± 0.78
Patient	3.23 ± 0.9
**PMU total score (mean ± SD)**	**64.88 ± 26.91**
Control	67.09 ± 29.24
Patient	62.65 ± 24.28
**Patient group epilepsy status (n, %)**	**91 (100%)**
Not having epilepsy	40 (43.96%)
Having epilepsy	51 (56.04%)
**Patient group perception (need for support) status (n, %)**	**91 (100%)**
No special education	60 (65.93%)
Receiving special education	31 (34.07%)
**Patient group mobility status (n, %)**	**91 (100%)**
Mobile	59 (64.84%)
Assisted mobile	12 (13.19%)
Immobile	20 (21.98%)
**Patient group involvement status (n, %)**	**91 (100%)**
Tetraparesis	18 (19.78%)
Diparesis	29 (31.87%)
Hemiparesis	44 (48.35%)

n: number of patients, mean ± SD: mean ± standard deviation.

**Table 2:** Comparison of the patient group and control group in terms of age, gender, sleep duration, and screen exposure.

	Control	Patient	*p*
	Group	Group	
**Female**	28; (%49.1)	29; (%50.9)	***p* ^a^** = 0.834
**Male**	64; (%50.8)	62; (%49.2)	
**Age (year)** **(mean ± SD); (median)**	81.85 ± 22.9; (84)	80.65 ± 31.51; (72)	***p* ^b^** = 0.271
**BMI SDS (mean ± SD)**	0.13 ± 0.02	0.12 ± 0.01	***p* ^c^** = 0.78
**Sleep duration (h) (mean ± SD); (median)**	9.4 ± 1.09; (10)	9.66 ± 0.91; (10)	***p* ^b^** = 0.116
**Screen exposure (h) (mean ± SD); (median)**	2.71 ± 0.78; (3)	3.23 ± 0.9; (3)	***p* ^b^** = 0.000

^a^ Chi-square test; ^b^ Mann–Whitney U-test; ^c^ Independent samples *t*-test.

**Table 3:** Comparison of problematic media use questionnaire scores among groups.

	Problematic Media Use Questionnaire Score	*p*
**Control group (mean ± SD); (median)**	67.09 ± 29.24; (60.5)	*p* ^a^ = 0.425
**Patient group (mean ± SD); (median)**	62.65 ± 24.28; (60)	
**Receiving special education (mean ± SD)**	60.00 ± 24.14	*p* ^b^ = 0.815
**No special education (mean ± SD)**	64.02 ± 24.44	
**Having epilepsy (mean ± SD)**	65.10 ± 25.40	*p* ^b^ = 0.322
**Not having epilepsy (mean ± SD)**	59.53 ± 22.70	
**Tetraparesis (mean ± SD)**	63.56 ± 26.4	*p* ^c^ = 0.978
**Diparesis (mean ± SD)**	62.83 ± 26.13	
**Hemiparesis (mean ± SD)**	62.16 ± 22.65	
**Mobile (mean ± SD)**	62.63 ± 24.77	*p* ^c^ = 0.568
**Assisted mobile (mean ± SD)**	56.75 ± 20.06	
**Immobile (mean ± SD)**	66.25 ± 25.51	

^a^ Mann–Whitney U-test; ^b^ Independent samples *t*-test; ^c^ One-way ANOVA test; mean ± SD: mean ± standard deviation.

## Data Availability

The data presented in this study are available on request from the corresponding author.

## Abbreviations

AAP	American Academy of Pediatrics
BMI	Body mass index
CP	Cerebral palsy
PMU	Problematic media use
